# The Relationship between Fatigue in Mothers and the Age of Their Less-Than-24-Month-Old Newborns

**DOI:** 10.3390/ijerph18126590

**Published:** 2021-06-18

**Authors:** Mar Sánchez-García, María José Cantero, Pedro M. Valero-Mora

**Affiliations:** 1 Departamento de Psicología Evolutiva y de la Educación, Universitat de València, Blasco Ibáñez, 21, CP:46010 Valencia, Spain; mmsanche@uv.es (M.S.-G.); maria.j.cantero@uv.es (M.J.C.); 2 Departamento de Metodología de las Ciencias del Comportamiento, Universitat de València, Blasco Ibáñez, 21, CP:46010 Valencia, Spain

**Keywords:** motherhood, fatigue, infant

## Abstract

The birth of a child marks the beginning of a new developmental period for the parents. These changes have positive but also negative aspects, such as the increase in fatigue experienced by mothers during this period of time, which can be very limiting and lead them to postpone other life or work goals. However, despite the widespread nature of this problem, there is a lack of information about the duration of this fatigue, with estimates ranging from three months to six years; this prevents mothers from planning this period of their lives adequately. The current study evaluated fatigue in a Spanish sample of 67 women with infants less than two years old and drew a comparison with another sample of 46 women with similar characteristics who were not caring for an infant at that time in order to determine the differences between them and how fatigue in the former group evolves depending on the age of the infant. The results show that fatigue is effectively maintained until at least two years after the birth of the infant.

## 1. Introduction

The birth of a child marks the beginning of a new stage of life for both parents, and especially for the mother. During the first several months after giving birth, mothers experience a large number of psychological, physiological and behavioral changes; although some of these are known, some aspects still need to be investigated in depth. Studies into the health of mothers after the birth of an infant indicate a high incidence of various health problems [1,2,3,4,5]. O’Hara and Wisner [6] define perinatal mental illness as a group of psychiatric disorders that are prevalent during pregnancy and can last as long as 1 year after delivery. They consider postpartum depression, anxiety disorders and psychosis as the most serious problems, but they also mention the so-called "baby blues", which typically lasts for a few days after the infant is born and has been estimated to affect 26% to 84% of mothers; in addition, it can sometimes develop into an episode of postpartum depression. Another problem that is not usually considered a psychiatric problem but lasts longer than the "baby blues" is fatigue, which is a complex, common, poorly studied and poorly understood problem that affects mothers and their families [2,7], and its effects can persist for a long time, beyond the first year postpartum [7]. Fatigue persists beyond the traditional six weeks after delivery, also known as "quarantine", and can have far-reaching consequences for the wellbeing of both mothers and their families.

According to McQueen and Mander [8], tiredness and fatigue affect individuals’ physical and mental abilities, causing carelessness, forgetfulness, irritability, a lack of physical energy and reduced concentration. Likewise, different authors have highlighted that fatigue interferes with the cognitive, emotional and psychomotor functioning of the sufferer [9,10,11]. Fatigue can have serious consequences for daily living, accident prevention and performance [12]. Several studies also link fatigue with postpartum depression [13,14,15].

Evidence-based interventions for the management of postpartum fatigue are scarce [3,16], although it is important for women to know that postpartum fatigue is highly likely to occur and that they should seek help without feeling guilty about their level of exhaustion [16]. Among the factors that affect mothers’ fatigue are daily chores [17]. Thus, the greater demands of day-to-day life during this vital period of time, and the fewer opportunities that mothers have to take breaks, appear to be responsible for the fatigue among them [18]. For example, in Sinai and Tikotzky [19], it was shown that the combination of fragmented sleep in mothers, especially in the case of those who were on maternity leave, and the constant need to care for the infant, with few opportunities to rest during the day, caused them to experience high levels of stress.

One important issue that must be addressed is the duration of the fatigue suffered by mothers during the first few months of raising the infant. This fatigue is, in part, a product of the lack of sleep associated with the infant’s feeding, care and sleep rhythm—since the newborn’s sleep rhythms are not yet well-established and their needs for food, affection, cleanliness and activity are not synchronized with the rhythms of the parents—and also with other factors that can cause the fatigue to become chronic [20] and evolve into other, more serious problems such as chronic stress or depression [21,22]. Thus, although mothers’ may sleep for longer once the infant matures, the pressure of the abovementioned tasks—which had been postponed because they were regarded as less urgent—is still present, delaying the recovery from fatigue beyond the first few months. It is known that when positive expectations regarding the experience of motherhood are not met, the result is a feeling of frustration that can aggravate the problems of depression and stress previously mentioned. However, since information about the duration of fatigue is usually obtained mostly from informal sources, such as friends, family or other parents with young children, it could often be biased in an optimistic way and lead to unrealistic expectations for the mothers. For this reason, the objective determination of the typical duration of this period could be very valuable to individuals undergoing this stage of life.

In order to evaluate fatigue, it is necessary to define it and to determine how to measure it. The definition of fatigue has long challenged scientists [23,24]. Some examples are Libbus et al. [25]’s definition as “the subjective report of exhaustion and decreased capacity for both physical and/or mental activity”; the North American Nursing Diagnosis Association’s definition as “the self-recognized state in which an individual experiences an overwhelming sustained sense of exhaustion and decreased capacity for physical and mental work that is not relieved by rest” [26]; and Shen et al. [27]’s definition: “fatigue is an overwhelming sense of tiredness, lack of energy and a feeling of exhaustion, associated with impaired physical and/or cognitive functioning”.

Given the difficulty of defining fatigue, Aaronson et al. [23] suggest that a definition or conceptual framework must “recognize the contributions of physiological and psychological functioning as well as social and cultural factors on the experience of fatigue”. Thus, they define fatigue as “the awareness of a decreased capacity for physical and/or mental activity due to an imbalance in the availability, utilization, and or restoration of resources needed to perform activity”. This definition assumes that fatigue has a subjective nature and consequently requires awareness of the phenomenon. The term “resources” is deliberately wide, as they may include biological, physiological, social and cultural factors that affect how one interprets one’s fatigue. Fatigue is the result of an imbalance between the utilization and the restoration of resources, “either because the demand or need is too great or because mechanisms of utilization and restoration are disturbed” [23]. This definition supports the idea that discrepancies between expectations of functioning and real functioning can affect the perception of a resource imbalance and lead to a possible line of intervention focused on the adjustment of expectations.

The definition above states that fatigue is mainly subjective, and, according to [27], “… there is no objective tool for measuring fatigue. It has been proposed that ‘fatigability’ is an objective inability to sustain power, which can be measured by electrophysiological methods, but to date, attempts to objectively measure fatigue have failed”. The review of instruments carried out by Whitehead [28] found 22 measures that fulfilled, to varying degrees, the criteria for being an “ideal” measure and 17 additional measures that only met some of the criteria. A few of these measures showed good properties and were recommended in her paper; however, as she also mentions, it is of importance to consider instruments that have been used among the populations of interest in order to ensure that the aspects of fatigue considered are adequate. Of these, the Visual Analogue Fatigue Scale (VAS-F) is regarded as adequate for our purposes because it has been validated in studies into mothers’ fatigue [29,30,31,32]. However, as mentioned above, fatigue in mothers usually occurs over long periods of time and VAS-F focuses on the specific moment at which the individual is evaluated, so that it does not satisfactorily assess whether this state has been occurring for a long period of time. For this reason, the Fatigue Assessment Scale (FAS) [33,34], which is the only measure mentioned by Whitehead [28] as having an assessment time frame longer than a few weeks, was also used in this study. These two measures will be described more thoroughly in the Section 2.

It should be noted that this paper assumes that fatigue and sleepiness are distinct phenomena that are better discussed separately. Thus, although fatigue is commonly associated with sleepiness, their implications in terms of diagnosis and treatment are different; despite this, these terms are often used interchangeably or merged under the general term of “tired” [27]. The interaction between sleepiness and fatigue seems to be important among women during the postpartum period [29], in the sense that wakefulness, or, rather, a lack of sleep, can increase fatigue, and this, in turn, can increase sleepiness. These constructs are related even though the associated risk factors are different [35]. In general, it is assumed that sleepiness is caused by reduced or altered sleep, which is, therefore, a necessary and sufficient requirement; meanwhile, fatigue can also be caused by reduced or altered sleep, but other causes are also possible [27].

Due to the differences between fatigue and lack of sleep, they are justifiably studied separately. Since both constructs tend to be confused and a lack of sleep often does resolve itself as the infant matures, there is a tendency to believe that the same is true for fatigue, despite the differences between them. For example, a lack of sleep often improves in the short term simply after a sufficient period of sleep, but fatigue can prevent the sufferer from falling asleep, or it may not resolve itself even after a period of sleep.

The following study aims to establish the relationship between fatigue in mothers and the age of their infants until they are two years old, and to evaluate whether their fatigue differs from that of women with similar characteristics but who are not caring for an infant. As the common assumption is that the fatigue is only present during the first few months after the child’s birth, an important aspect to evaluate is whether there are differences in fatigue for mothers with infants over 6 months old up to two years old. In this case, fatigue would occur despite the fact that the mothers are able to recover the hours of sleep that were lost when the infant was younger.

## 2. Design and Method

In this study, a sample of 113 women was used, 67 of whom were caring for a dependent infant under 2 years of age, while 46 were not. The women were interviewed about a series of relevant variables related to the fatigue that they experienced. The responses were evaluated to test the hypothesis that women caring for infants had higher levels of fatigue compared to women who were not caring for infants and that this fatigue lasted beyond the first semester of the newborn’s life.

The design of this study corresponds to the description in Shadish et al. [36] of a quasi-experimental post-test-only study. Two groups (one treatment group and one control group) took part in the study, which was retrospective and questionnaire-based. The study required the two groups of women to complete the questionnaires described in the Section 2.2. Prior to the procedure, written consent was obtained from all of the participants for the aggregate use of the results for research purposes. This study was conducted following the guidelines set out by our institution. As the data collection was carried out as part of a larger study involving other tests and procedures, the participants were individually invited to a quiet room located in our facility, where they were given the forms to complete and pencils. One of the authors of the article was present at the time to help the participants and ensure that they could work without interruption, as some of them brought their infants to the laboratory.

The participants were not provided any additional information on the study’s objectives before their answers were registered. Once they had finished, the objective of the study was succinctly explained to them and they were compensated for their participation (EUR 30).

### 2.1. Participants

Women caring for infants and willing to participate in a study into maternal fatigue were contacted in the waiting room of a pediatrician’s office. The requirements were that they were between 25 and 50 years old and did not have any serious health problems. Moreover, their children had to be between 1 and 24 months old.

Women who were not caring for infants under 24 months old were contacted first in a pediatrician’s waiting room but also through the friends, family and co-workers of the women who had already participated in the study. The criteria for inclusion in this group were the same as for the first group, except that they were required not to have children or their children had to be at least 6 years old.

The total number of participants in the study was 113 women, of whom 67 met the criteria to be classified within the group of those taking care of children. Henceforth, we refer to this sample as "with infants" (for the women caring for infants). In turn, the other 46 met the criteria to be classified as those not caring for infants and we refer to them as "control" (for the women not caring for infants).

### 2.2. Measurements

The two measurements of fatigue used in this study were the Fatigue Assessment Scale (FAS) and the Visual Analogue Scale to Evaluate Fatigue. These are described below:

- Fatigue Assessment Scale (FAS) [34]: This is a 10-item scale that evaluates symptoms of chronic fatigue. It comprises two subscales, one related to physical symptoms and the other to mental symptoms of fatigue, although, for this study, they were combined in a general measure. FAS is a self-administered paper and pencil scale that takes around 5 min to complete. The authors found a Cronbach alpha of 0.90. Some studies that have used this scale are: Cooklin et al. [37], Dunning et al. [38] and Giallo et al. [3]. This scale was originally validated in a population of men and women with mean age and standard deviation of 45 ± 8.4 years and 43 ± 9.5 years, respectively. Each of the items is answered using a five-point Likert-type scale, where 1 = never and 5 = always. Total scores range from 10, which indicates the lowest level of fatigue, to 50, which corresponds to the highest level of fatigue. The internal consistency coefficient (Cronbach’s alpha) in the sample of this study was 0.86.
- Visual Analogue Scale to Evaluate Fatigue Severity [39]: This scale consists of 18 items related to the subjective experience of fatigue. For each item, respondents should mark with a circle or an “X” along a visual analogue line that runs between two extremes (for example, from “not at all tired” to “extremely tired”), reflecting how fatigued they feel at that moment. The scale has been validated with adults, men and women, aged between 18 and 55 years old. It is a pencil and paper self-assessment scale, which requires an approximate time of 5–10 min to perform. The psychometric evaluations carried out by Lee et al. [39] have shown a high internal reliability that ranges from 0.94 to 0.96. The scale has two subscales, namely fatigue (VAS FATIGUE) and energy (VAS ENERGY), which will be reported separately in this study and which are negatively correlated. On the fatigue subscale, high scores indicate a high level of fatigue. On the energy subscale, high scores indicate a high energy level. This scale has been used in the following studies among others: Elek et al. [40], Pugh et al. [10], Troy and Dalgas-Pelish [41] and Waters and Lee [31]. The internal consistency coefficient (Cronbach’s alpha) in the sample of this study was 0.95.

Additionally, the mothers were asked about the total number of hours of sleep that they had managed to achieve in the week in which the study was carried out, counting both the nighttime period and any naps throughout the day.

### 2.3. Data Analysis

For data analysis purposes, the scores from the scales were added up after inverting the negative items. The data were analyzed graphically (scatter and box plots) and using *t*-tests and correlations. Significance was always evaluated at *p* < 0.5. All of the calculations were done and the graphs plotted using R [42,43]. There was a very small number of missing values, but given that the analyses were exclusively univariate, the impact was very limited.

**Figure 1:**
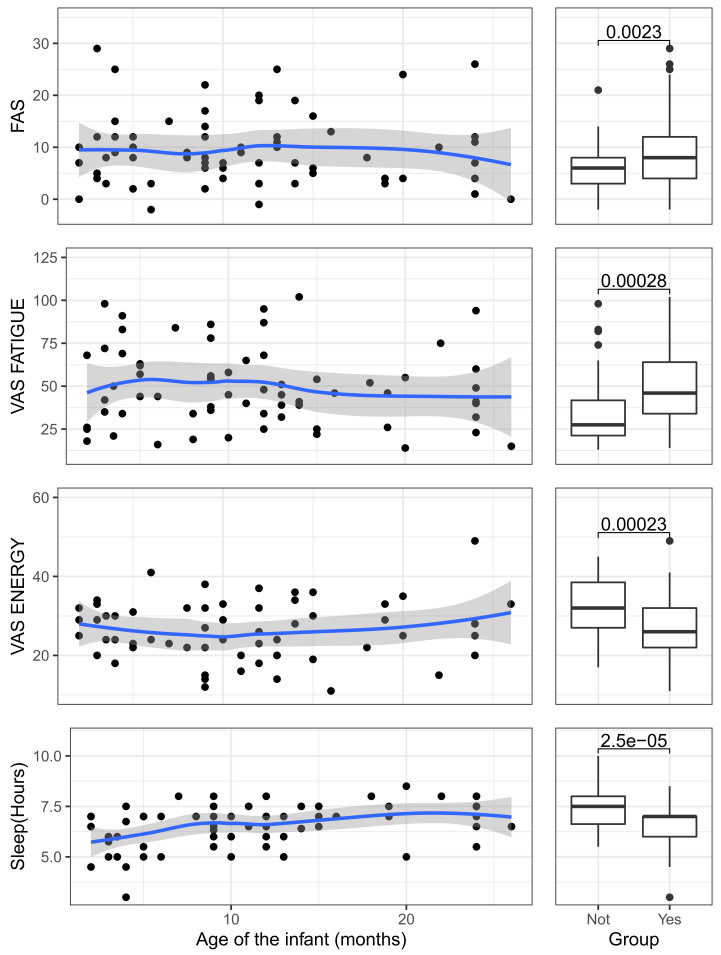
Scatterplots showing the relationship between the age of the infants and the variables of fatigue/hours of sleep of their mothers, and boxplots showing the comparison between the levels of fatigue/hours of sleep of women with or without infants.

The relationship between the age of the infant and the indicators of fatigue and sleepiness are displayed graphically in Figure 1. This figure has a couple of plots per indicator, with a total of eight indicators corresponding to the three sleepiness scales plus the answers to the questions evaluating nighttime sleep. The first plot of each couple is a scatterplot in which the age of the infant is set in the horizontal axis and the indicator is set in the vertical axis. As the relationship between the age of the infant and the indicators was found to be non-linear in many cases, we overimposed a non-parametric loess curve in all the plots to better visualize the relationship between the variables [44]. The second plot of the couple is a boxplot for comparing the two groups of women in our study: the control group (mothers without infants under 6 years old at the time of the study) and mothers with infants. Each boxplot shows overimposed the result of a t-test comparing the means of the groups in each indicator. As the scatterplots and the boxplots share the same vertical axis, it is possible to assess when the values of the scatterplot are above or below the median values of the indicators for the two groups of mothers.

## 3. Results

### 3.1. Sample Description

Table 1 shows a comparison between the two samples of women considered in the study. Here, we can see that the age of the two samples was similar. In the group of women without infants, the percentage of married or equivalent women was somewhat lower than in the group with infants. The percentage of unemployed women (and therefore its opposite, those with a job at the time of the study) was similar. Finally, the two samples of women were generally free of health problems, except for one case, but a detailed analysis of this led us to include it since the reported problem did not affect the participant’s daily functioning.

### 3.2. Differences in Fatigue and Sleepiness

The right-hand panels of Figure 1 show the comparison of the means of the participants with or without infants in their care on the scales used in the study, as well as the number of hours for which they slept regularly during the week that the study was carried out. In every case, it is possible to see that the differences are significant, with the mothers caring for infants suffering greater chronic fatigue according to the FAS (ΔM=3.35, 95% CI [1.23, 5.48], t(110.52)=3.12, p=0.002), greater acute fatigue according to the VAS FATIGUE measure (ΔM=15.34, 95% CI [7.25, 23.43], t(104.67)=3.76, p<0.001), and lower energy according to the VAS ENERGY measure (ΔM=−6.10, 95% CI [−9.04, −3.16], t(100.10)=−4.12, p<0.001). Furthermore, the mothers who had infants obtained approximately one hour less sleep compared to those who did not at the time of the study (ΔM=−0.82, 95% CI [−1.19, −0.45], t(106.45)=−4.41, p<0.001).

The above results are to be expected in the sense that they confirm the well-established fact that mothers suffer significantly more fatigue than those without infants during the time for which they care for their infants and that, in addition, they do not obtain a similar number of hours of sleep. However, these data do not show the evolution of fatigue depending on the age of the infant or whether their situation improves as the infant matures. To analyze this, the next section will examine measures of fatigue and hours of sleep in mothers based on the age of the infant.

### 3.3. The Relationship between Mothers’ Fatigue and Infants’ Age

Figure 1 comprises several panels that show two graphs for each fatigue indicator: (a) a scatterplot showing the relationship between the age of the infants and the variables of fatigue/hours of sleep of their mothers, and (b) a boxplot showing a comparison between the levels of fatigue/hours of sleep of women with and without infants. The variable to which each panel refers is indicated on the abscissa axis of the scatterplot. The ordinate axis always shows the infant’s age but this label is only shown in the last two panels to avoid repetition. In addition, a loess curve [44] was added to the scatterplots to visualize the possible curvilinearity of the relationship between age and fatigue. Finally, the boxplot superimposes the result of the t-test for the comparison of means between the group of women currently not caring for infants (No) and those caring for them (Yes) for each of the variables. This arrangement makes it possible to compare the evolution of the variables of fatigue/hours of sleep with the values that we could consider normal, taken from mothers without infants.

The results show that the three variables follow a similar trend. FAS, an indicator of chronic fatigue, runs at practically the same level during the period of time considered in this study and only seems to decrease slightly when the infants are approaching 30 months old. The acute fatigue measured by VAS FATIGUE and by VAS ENERGY also remains stable, although both measures show a tendency to recover towards the end of the series.

The loess lines overimposed on the scatterplots of Figure 1 show that the relationships do not deviate strongly from linearity, so they can be summarized using linear correlations as those shown in Table 2. In this table, it may be observed that, in accordance with the initially established hypotheses, the correlations between the fatigue variables for these mothers and the age of their infants are not significant, unlike the amount of total sleep, which does correlate significantly with the age of the infants at the time of the study. We also include the correlation between the age of the mother and the previous variables as this might be a possible confounding factor in the analysis. The age of the mother correlated significantly with the age of the infant but it did not correlate with the fatigue and sleep variables.

## 4. Discussion

The results show that mothers caring for infants experience greater fatigue than women of similar characteristics who are not caring for an infant. More importantly, fatigue does not decrease even if the infants are older; rather, it remains similar for mothers with infants up to 26 months of age. It is important to note that this stability occurs even when it can be observed that the number of hours of sleep that mothers enjoy increases as the infant grows older, which supports the hypothesis that the cause of mothers’ fatigue is related to factors other than a mere lack of rest.

The above results are compatible with the notion that, during the first few months of their infants’ lives, mothers accumulate fatigue due to the increase in the number of tasks that they must fulfil in addition to the lack of sleep. Since the demands of these tasks, related to life, academic or work goals, exceed their capacities and resources, the strategy that mothers probably follow is to temporarily delay their completion until their child is older. However, when this happens and infants begin to gain autonomy, offering relief to their mothers, the number of tasks remaining possibly still represents a significant challenge, so that the mothers cannot be given the amount of rest necessary to alleviate their fatigue.

The present study is subject to several limitations, which we comment on below. Firstly, neither the size of the sample used nor the collection method allow us to generalize to the reference population, so this work should be considered as a pilot study that fundamentally serves to lay the foundations for a further study. Secondly, the conclusions obtained here would be strengthened by a study based on a longitudinal design, in which the same mothers indicate their levels of fatigue over various time points. Thirdly, it would be appropriate to extend this study to the rest of the members of the family unit, to assess their level of fatigue as well as possible strategies to combat the problem in a comprehensive way.

The overall conclusion of this study is that the perception of fatigue in mothers with infants is higher than in women without children and that it remains at the same level independently of their infant’s age (within a range of 0 to 24 months old). Contrary to the usual assumption, fatigue is not related to the amount of sleep of the mothers, so, although having older children generally means more sleep for mothers, their fatigue is not alleviated. This study did not analyze factors that might explain why the fatigue does not subside but some possible explanations are misadjustment of expectations, job status, economic changes, lack of family support and having more children.

## Figures and Tables

**Table 1:** Descriptive statistics for the two samples of women that participated in the study. Group = Yes were mothers who were taking care of infants under 24 months old at the time of the study and Group = Not were women without children under 6 years.

Variables	N		Group = Not	Group = Yes	Total	*p*
Total N (%)			46 (40.7)	67 (59.3)	113	
Age	111	Mean (SD)	36.9 (7.9)	34.6 (5.1)	35.5 (6.5)	0.061
Married or equivalent relationship	111	0	10 (22.2)	4 (6.1)	14 (12.6)	0.026
		1	35 (77.8)	62 (93.9)	97 (87.4)	
Unemployed?	113	0	9 (19.6)	17 (25.4)	26 (23.0)	0.622
		1	37 (80.4)	50 (74.6)	87 (77.0)	
Currently employed or maternity leave	88	0	19 (51.4)	17 (33.3)	36 (40.9)	0.001
		1	18 (48.6)	19 (37.3)	37 (42.0)	
		2	0 (0.0)	15 (29.4)	15 (17.0)	
Health problems	112	0	0 (0.0)	1 (1.5)	1 (0.9)	1.000
		2	45 (100.0)	66 (98.5)	111 (99.1)	
Infant’s age	67	Mean (SD)		11.4 (6.9)	11.4 (6.9)	
Age another child	87	Mean (SD)	12.4 (7.7)	12.6 (10.0)	12.6 (9.5)	0.909
Count of another child	113	Not	24 (52.2)	2 (3.0)	26 (23.0)	
		Yes	22 (47.8)	65 (97.0)	87 (77.0)	
Age of yet another child	35	Mean (SD)	10.5 (7.0)	7.1 (5.0)	8.5 (6.1)	0.105
Count of yet another child		Not	31 (67.4)	47 (70.1)	78 (69.0)	
		Yes	15 (32.6)	20 (29.9)	35 (31.0)	

Note: *p* value corresponds to a mean difference *t*-test or a chi-squared test.

**Table 2:** Correlations between the three fatigue scales (FAS, VAS FATIGUE and VAS ENERGY), sleep (hours per night) and the age of their infants, for the participants.

	Infants’ Age	FAS	VAS FATIGUE	VAS ENERGY	Mothers’ Sleep
Infant’s age					
FAS	−0.04				
VAS FATIGUE	−0.12	0.68 ***			
VAS ENERGY	0.04	−0.45 ***	−0.47 ***		
Mother’s sleep	0.40 ***	−0.16	0.01	0.10	
Mother’s age (years)	0.25 *	−0.10	−0.04	0.05	0.17

Note: * *p* < 0.05. *** *p* < 0.001.

## Data Availability

The data presented in this study are available on request from the corresponding author. The data are not publicly available due to resource limitations.
